# Fruit and vegetable consumption in adolescence and health in early adulthood: a longitudinal analysis of the Statistics Canada’s National Population Health Survey

**DOI:** 10.1186/1471-2458-13-1206

**Published:** 2013-12-20

**Authors:** Yuriko Takaoka, Norito Kawakami

**Affiliations:** 1 School of Integrated Health Sciences, Faculty of Medicine, The University of Tokyo, 7-3-1 Hongo, Bunkyo-ku, Tokyo 113-0033, Japan; 2 Department of Mental Health, Graduate School of Medicine, The University of Tokyo, 7-3-1 HongoBunkyo-ku, Tokyo 113-0033, Japan

**Keywords:** Fruit and vegetable consumption, Self-rated health, Mental health, Adolescence, Early adulthood

## Abstract

**Background:**

The present study aimed to explore a longitudinal relationship between fruit and vegetable consumption in adolescence and two health-related outcomes (i.e., self-rated health and mental health) in early adulthood in the community.

**Methods:**

Data from a longitudinal cohort of the Canadian National Population Health Survey (NPHS) were used. Participants of the 2002/03 survey aged 15-17 years old were followed and surveyed in 2008/09. The number of the sample used in the statistical analyses was 250 (n = 250). Multiple logistic regression analyses were used to assess the associations of fruit and vegetable consumption in the adolescence (classified into tertiles) with non-excellent (or poor) self-rated health and poor mental health (defined as having a K6 score of 5+) at follow-up.

**Results:**

After adjusting for sex, age, the highest level of education in household, and the other covariates, participants who consumed fruits and vegetables most frequently at baseline had a significantly smaller odds ratio for being non-excellent self-rated health (OR 0.30, 95% CI 0.11, 0.83). No significant associations were found between fruit and vegetable consumption at baseline and poor mental health at follow-up in any model (*p* > 0.05).

**Conclusions:**

The results of this longitudinal study suggest that high fruit and vegetable consumption in adolescence has a beneficial influence on self-rated health in the early adulthood.

## Background

It is well established that a healthy diet is a key factor in preventing chronic diseases and maintaining health throughout the life course [1]. Low consumption of fruits and vegetables was ranked as the 5th cause of disability-adjusted life year in the most recent Global Burden of Disease 2010, respectively [2].

High consumption of fruits and vegetables has been associated with reduced risk of chronic diseases such as heart disease, diabetes, and cancer [3-7], as well as increased health-related quality of life [8]. In addition, using self-rated health as a comprehensive indicator of health [9], previous studies have found positive associations between fruit and vegetable consumption and better self-rated health [10-13].

Regarding mental health, results are mixed. Some studies have shown that intake of fruits and vegetables, folate, or vitamin C positively affects mental health [14-18]. Other studies have shown inconsistent or no associations [8,19,20]. Only one longitudinal study conducted in a general population reported no significant association between fruit and vegetable intake and mental health [19].

Most previous studies have focused on adults or the elderly. However, much less is known about the relationship between fruit and vegetable consumption and self-rated physical and mental health in adolescence and early adulthood. Adolescence and early adulthood are important periods of life for two reasons. First, researchers suggest that dietary habits in childhood or adolescence tend to remain the same in adulthood [21-23]. Therefore, habits of fruit and vegetable consumption could have a longer, and thus, more significant impact on health over the lifetime. Second, physical and mental health in adolescence and early adulthood has a large impact on people’s lives [24]. In particular, the age of onset for many mental disorders is during these periods of life and could have a lasting impact on human capital during adulthood [24,25]. However, limited research has been presented on the association between fruit and vegetable consumption and health in adolescence. Two previous studies reported that fruit and vegetable consumption was related to better self-rated health in adolescence [26,27]. Other studies that have only focused on vegetable consumption also reported positive associations between these factors [28]. However, these studies were cross-sectional. It is important to investigate whether fruit and vegetable consumption could have a beneficial impact on physical and mental health in these key stages of life by using a longitudinal study design. Once established, such an association could have implications for health policy by providing evidence to encourage fruit and vegetable consumption in adolescence to promote physical and mental health in early adulthood.

The present study aimed to explore the longitudinal relationship between fruit and vegetable consumption in adolescence and two health-related outcomes (i.e., self-rated health and mental health), in early adulthood using population-based cohort data from Canada [29].

## Methods

### Data sources

The current analyses were based on data from the Statistics Canada’s National Population Health Survey (NPHS), which began in 1994/95 [29]. The initial surveys employed a stratified multi-stage sampling design and covered private household and institutional residents in all provinces except residents of Indian reserves, Canadian Forces bases, and some remote areas. The first three cycles (1994/95 to 1998/99) have both longitudinal and cross-sectional components. Starting in 2000/01 (cycle 4), the NPHS became a solely longitudinal study. The longitudinal sample was composed of 17,276 respondents (8,046 males, 9,230 females) 12 years of age or older who were followed every two years over time (i.e., cycle 5 in 2002/03, cycle 6 in 2004/05, cycle 7 in 2006/07, and cycle 8 in 2008/09). More detailed description of the NPHS design, sample, and interview procedures can be found in published reports [29].

This study used part of the NPHS longitudinal data (i.e., data collected in cycle 5 (2002/03) for a baseline and cycle 8 (2008/09) for a follow-up) because information on fruit and vegetable consumption was not available earlier than cycle 5 (2002/03). We restricted our sample to adolescents aged 15–17 years old at baseline in cycle 5 (2002/03). Those who completed questions on relevant variables in this study (health outcomes at baseline and follow-up, fruit and vegetable consumption at baseline, and other covariates at baseline) were regarded as the study sample (n = 383). The response rate was 80.8% for cycle 5.

The NPHS data were available for any researcher for analysis. The direct access to the data was limited within Canada. However, Statistics Canada allows researchers outside of Canada to analyze the data by “remote access.” After obtaining permission to analyze the NPHS data from Statistics Canada, we received dummy data, for which we created SAS programs for the analysis. Statistics Canada conducted the analysis using the program on the data server with the actual data and sent the results to us. The process was conducted about 16 times from September 2012 to September 2013 to obtain the final results. Informed consent from participants was obtained by Statistics Canada. This study was approved by the Research Ethics Committee of the Graduate School of Medicine/Faculty of Medicine, the University of Tokyo.

### Methods

1) Fruit and vegetable consumption

Fruit and vegetable consumption at baseline in cycle 5 (2002/03) was evaluated as the total number of times per day that a participant ate fruits and vegetables [30]. This measure was assessed with six questions following the instruction: “Think about all the foods you eat, both meals and snacks, at home and away from home.” The six questions were as follows:

1. How often do you usually drink fruit juices such as orange, grapefruit, or tomato? (e.g., once a day, three times a week, twice a month)?

2. Not counting juice, how often do you usually eat fruit?

3. How often do you usually eat green salad?

4. How often do you usually eat potatoes, not including French fries, fried potatoes, or potato chips?

5. How often do you usually eat carrots?

6. Not counting carrots, potatoes, or salad, how many servings of other vegetables do you usually eat?

For each item, respondents were asked to report the frequency of consumption either in a day, week, month, or year. Reported frequencies were converted into frequency per year, and then summed up for all six items, divided by 365 to derive an aggregate of the daily frequency of fruits and vegetables consumed. This questionnaire has been validated as a proxy for quantified intake of vegetable and fruit consumption in the community populations in the United States and Canada [31,32]. For the analysis, two types of classification of fruit and vegetable consumption were used: (a) tertiles based on the distribution of participants’ scores. i.e., those whose score was 3.14 or less (low), those whose score was more than 3.14 and 5 or less (moderate), and those whose score was more than 5 (high); (b) dichotomization into those who had a score of five or more (high) and those who had a score of less than five (low), using the minimum recommended in Canada [33].

2) Outcome variables

Two outcome variables, self-rated health and mental health, were measured at baseline in cycle 5 (2002/03) and at follow-up in cycle 8 (2008/09).

(1) Self-rated health

Numerous studies have shown that self-rated health is a valid indicator of current health and an independent predictor of chronic diseases and premature death [9]. A single question is often used to measure self-rated health and responses have been shown to be positively correlated with clinical assessments [34,35]. In this study, self-rated health was measured by a single question “In general, would you say your health is: excellent, very good, good, fair, or poor”. For the analysis in this study, two types of classification of self-rated health were used in consideration of the distribution of the variable, (a) classify respondents into those with excellent health (upper 25%) and non-excellent health (very good, good, fair, and poor) (bottom 75%) or (b) classify respondents into those with better health (excellent and very good) (upper 75%) and poor health (good, fair, and poor) (bottom 25%).

(2) Mental health

Mental health was measured using the K6 scale [36,37]. The K6 scale is a 6-item scale used to quantify non-specific psychological distress. The minimum score is 0 and the maximum score is 24, with a higher score being indicative of greater psychological distress (e.g., depression and anxiety). For this analysis, participants were divided into two groups, K6 score ≤ 4 and K6 score ≥ 5. The cut-off was adopted based on a recent study in Canada [38].

3) Other variables

(1) Socioeconomic position

Childhood and adolescent socioeconomic position (SEP) is a strong predictor of health in adulthood [39,40]. Two indicators for measuring SEP at baseline were used in this study, the highest level of education in the household and household income. The highest level of education in the household was defined as the highest education completed by the father or mother in the household, was only measured in cycle 3 (in 1998/99), and was based on interviews with both respondents and respondents’ families four years before the baseline. Because the variable was less likely to change within the same household, we used this variable as a SEP indicator at baseline. Participants were classified into three groups, secondary school graduation or less (low), some post-secondary (middle), and post-secondary graduation (high). Household income was assessed by the total household income of all sources over the previous 12 months based on interviews with respondents at baseline in cycle 5 (2002/03). For the analysis, based on the distribution of the variable, participants were divided into four groups, $39,999 or less (low), $40,000 to $59,999 (lower middle), $60,000 to $79,999 (upper middle), and $80,000 or more (high). Unfortunately, about one-third of the respondents had a missing value on this variable. Thus, the analysis using household income was conducted among a limited number of respondents.

(2) Lifestyle-related variables

Several lifestyle-related variables were measured at baseline in cycle 5 (2002/03). Alcohol consumption of respondents at baseline was assessed as the average daily alcohol consumption in the week prior to the interview. For this analysis, participants were classified into a current drinker or not a drinker. Smoking status was assessed as the type of smoker based on respondents’ smoking habits. For this analysis, participants were classified into either a current smoker or not a smoker. Body mass index (BMI) was calculated by dividing weight in kilograms by height in meters squared. For this analysis, participants were split into two groups, BMI of 24.9 or less and BMI of 25.0 or more [41]. Physical activity was measured based on total daily energy expenditure (EE) (kcal/kg/day) for all activities in a day. The EE values are calculated as follows:

$$\mathrm{EE}\left(\text{kcal}/\mathrm{kg}/\text{day}\right)=\text{Sum of}(\left({\text{Ni}}^{*}{\text{Di}}^{*}\text{METvalue}\right)/365)$$

where Ni = the number of times a respondent engaged in an activity over a 12 month period; Di = the average duration in hours of the activity; and MET = the energy cost of the activity expressed as kilocalories expended per kilogram of body weight per hour of activity (kcal/kg per hour)/365 (to convert yearly data into daily data). For this analysis, participants were classified into two groups, inactive (EE less than 1.5) and moderate or active (EE of 1.5 or greater).

(3) Other variables

Sex and age in years at baseline were also used in the analysis. Household size at baseline was measured as the number of people living within a household. Participants were divided into tertiles and assigned a score ranging from 1 to 3; three or less people in a household (1), four people in a household (2), five or more people in a household (3).

### Statistical analysis

To estimate the association between fruit and vegetable consumption at baseline and self-rated health or mental health at follow-up, we performed a series of multiple logistic regression analyses. In the model 1, sex, age, and frequency of fruit and vegetable intake at baseline were entered into the equation. In model 2, a SEP indicator (the highest level of education in the household or household income at baseline) was additionally adjusted. Finally, in model 3, alcohol consumption, smoking status, BMI, physical activity, and household size at baseline were additionally adjusted. Odds ratios and 95% confidence intervals were estimated. *P-*values for group difference were estimated using Chi-square test. *P-*values for trends across categories of fruit and vegetable consumption were also calculated. A statistically significant *P-*value was set at 0.05. A sampling weight was created by Statistics Canada for each respondent in the NPHS data, and was based on a differential probability of participating in the study in terms of sex and age, follow-up rate, and their agreement to use the data for research. We considered the weights for every analysis using the Taylor series method implemented in the SURVEYFREQ, SURVEYMEANS, and SURVEYLOGISTIC procedures of SAS software version 9.3 (SAS Institute, Cary, North Carolina).

## Results

### Characteristics of participants

Among those aged 15–17 years old who participated in the study at baseline (N = 438), 383 completed all relevant variables for the analyses (except for household income). Among them, 250 successfully completed questions on self-rated health and mental health at follow-up. Table 1 shows weighted numbers and proportions of the final sample by baseline characteristics. Approximately half of the respondents were male. The baseline age was almost equally distributed to 15, 16, and 17-year-olds. Approximately 30% of participants had poor mental health (K6 scores ≥ 5), and approximately 75% of participants had non-excellent self-rated health. Respondents who were lost to follow-up were more likely males, older, less educated, smokers, obese, and having low fruit and vegetable consumption, compared with those who completed the follow-up. Among those who completed the follow-up, 165 also completed questions of household income; 19.7% had low income and 41.0% had high income. The number and proportion of respondents with middle-low and middle-high income were unknown because, according to the regulation of the Statistics Canada, numbers less than 30 were not reported. The other baseline characteristics were similar between the larger (250 respondents) and smaller (165 respondents) datasets (data available upon request).

**Table 1:** Baseline characteristics (weighted frequencies) of participants aged 15–17 years old for the final analysis selected from the Statistics Canada’s National Population Health Survey (NPHS)†

		**Final sample (n = 250)**
Characteristics at baseline (the cycle 5 in 2002/2003)	n	%
**Sex**		
Male	114	45.5
Female	136	54.5
**Age**		
15 years old	93	37.3
16 years old	87	35.0
17 years old	69	27.7
**Mental health (K6 scores)**		
< = 4	178	71.0
> = 5	72	29.0
**Self-rated health**		
Non-excellent (poor, fair, good, very good)	184	73.5
Excellent	66	26.5
**Fruit and vegetable consumption (summed frequency per day)**		
Low (< = 3.14)	78	31.3
Moderate (3.14-5.00)	84	33.7
High (5<)	88	35.0
**Current drinker (yes)**	161	64.4
**Current smoker (yes)**	29	11.5
**Physical activity**		
Active, Moderate	186	74.4
Inactive	64	25.6
**Household size**		
1,2,3persons	55	21.9
4 persons	113	45.1
5 persons and more	82	33.0
**Body mass index (BMI)**		
24.9 < =	209	83.5
> = 25.0	41	16.5
**Highest level of education in household**		
Low	25	10.1
Middle	60	24.1
High	165	65.8

†:Figures are rounded, thus the total number is not equal to the total number of respondents (n = 250).

A post-hoc statistical power (1-beta) was calculated as 0.631 to detect a double risk of poor health (or high psychological distress) (RR = 2.0) for the lowest tertile group in terms of fruit/vegetable consumption compared to the highest tertile group, assuming that a prevalence of poor health was 0.25, each tertile group included one third (n = 86) of the total sample, and alpha (p) was 0.05 (G*Power 3.1).

### Association of fruit and vegetable consumption and self-rated health

The upper half of Table 2 shows the results of the multiple logistic regression of self-rated health and mental health on the tertiles of fruit and vegetable consumption controlling for education, and the other variables among the 250 respondents. After adjusting for sex and age, participants who consumed fruits and vegetables most frequently at baseline had a significantly smaller odds ratio for being non-excellent on self-rated health (Model 1). This association was still significant after additionally adjusting for highest level of education in the household (Model 2) and the other covariates (Model 3). *P* for trend was also significant for all models (0.043, 0.021, and 0.020, respectively). Non-excellent self-rated health at baseline was significantly associated in models 1 and 2 (*p* < 0.05) and marginally significantly associated in model 3 (*p* = 0.053) with non-excellent self-rated health at follow-up. When an alternative classification of self-rated health (i.e., better health (excellent and very good) vs. poor health (good, fair, and poor)) was used, the frequency of fruit and vegetable intake was not significantly associated with poor self-rated health at follow-up (*p* > 0.05). In a fully adjusted model, the odds ratio (95% CI) of having poor self-rated health was 1.11 (0.41, 3.04) for those with moderate fruit and vegetable consumption, and 0.30 (0.11, 0.83) for those with high consumption (*p* = 0.013 for difference among the three groups).

**Table 2:** Association between fruit/vegetable consumption (Low, Moderate, High) at baseline (cycle 5 in 2002/03) and non-excellent self-rated health, poor mental health (K6 > = 5) at follow-up (cycle 8 in 2008/09) among respondents aged 15–17 years old (n = 250) from the Statistics Canada’s National Population Health Survey (NPHS)

	**Model 1**		**Model 2**		**Model 3**	
Controlling for:	Sex, age, and frequency of fruit and vegetable intake at baseline		Covariates controlled at Model 1+ highest level of education in the household		Covariates controlled at Model 2 + the other covariates† at baseline	
	OR	95% CI	OR	95% CI	OR	95% CI
Self-rated health						
Fruit and vegetable consumption = Low (< = 3.14)‡	1.00		1.00		1.00	
Fruit and vegetable consumption = Moderate (3.14-5.00)‡	1.16	(0.45-2.99)	1.16	(0.45-2.98)	1.11	(0.41-3.04)
Fruit and vegetable consumption = High (5<)‡	0.37	(0.14-0.98)	0.33	(0.13-0.85)	0.30	(0.11-0.83)
p for difference (df = 2)	0.033		0.011		0.013	
Mental health (K6 scores)						
Fruit and vegetable consumption = Low (< = 3.14)‡	1.00		1.00		1.00	
Fruit and vegetable consumption = Moderate (3.14-5.00)‡	1.04	(0.40-2.68)	1.03	(0.39-2.72)	1.22	(0.45-3.28)
Fruit and vegetable consumption = High (5<)‡	1.16	(0.46-2.98)	1.10	(0.42-2.87)	1.25	(0.45-3.45)
p for group difference (df = 2)	0.946		0.980		0.898	

OR, odds ratio; CI, confidential interval.

†:The other covariates included alcohol consumption, smoking status, BMI, physical activity, and household size.

‡:Frequency per day.

A series of similar analyses were conducted in a smaller sample (N = 165) of respondents for whom household income was available and that used household income instead of the highest level of education in household as a SEP indicator (data available upon request). Frequency of fruit and vegetable intake was not significantly associated with excellent or non-excellent self-rated health at follow-up (*p* > 0.05). In a fully-adjusted model (Model 3), the odds ratios (and the 95% CIs) of having non-excellent self-rated health were 2.41 (0.77, 7.54) for those with moderate frequency of fruit and vegetable intake and 0.63 (0.17, 2.32) for those with high frequency compared to those with low frequency (*p* = 0.104 for difference among the three groups). When the alternative classification of self-rated health was used, frequency of fruit and vegetable consumption was not significantly associated with poor self-rated health at follow-up (*p* > 0.05). In a fully-adjusted model (Model 3), the odds ratios (and the 95% CIs) of having poor self-rated health were 2.96 (0.88, 9.94) for those with moderate frequency of fruit and vegetable consumption and 0.86 (0.25, 2.94) for those with high frequency compared with those with low frequency (*p* = 0.090 for difference among the three groups).

### Association of fruit/vegetable consumption and mental health

No significant associations were found between fruit and vegetable consumption at baseline and poor mental health at follow-up when participants were divide into tertiles considering the distribution of their fruit and vegetable consumption scores: those whose score was 3.14 or less (low), those whose score was more than 3.14 and 5 or less (moderate), and those whose score was more than 5 (high) in any model (*p* > 0.05) (the bottom half of Table 2). *P* for trend was not significant in any model (0.748, 0.845, and 0.680, respectively). Good and moderate consumption of fruits and vegetables was associated with slightly greater odds ratios of having poor mental health at follow-up. Poor mental health at baseline and female sex were significantly associated with poor mental health at follow-up (*p* < 0.05).

In a series of similar analyses with a smaller sample (N = 165) of respondents for whom household income was available (data available upon request), frequency of fruit and vegetable consumption was not significantly associated with mental health at follow-up in any model (*p* > 0.05). In a fully-adjusted model (Model 3), the odds ratios (and the 95% CIs) of having poor mental health were 1.85 (0.56, 6.10) for those with moderate frequency and 1.57 (0.48, 5.13) for those with high frequency compared to those with low frequency (*p* = 0.581 for difference among the three groups).

### Association of fruit and vegetable consumption with self-rated health and mental health using the dichotomized categories

Table 3 shows the results of the multiple logistic regression of self-rated health and mental health on fruit and vegetable consumption controlling for parents’ education and the other covariates among the 250 respondents using the dichotomized grouping of participants based on the fruit and vegetable consumption score (n = 90 and n = 160 for high and low consumption groups, respectively). The patterns were almost similar: fruit and vegetable consumption was significantly and positively associated with excellent self-rated health. The association of fruit and vegetable consumption with metal health was not significant. In similar analyses with a smaller sample (N = 165) of respondents for whom household income was available, no significant association between fruit and vegetable consumption and self-rated health or mental health were found (data available upon request).

**Table 3:** Association between fruit/vegetable consumption (Low, High) at baseline (cycle 5 in 2002/03) and non-excellent self-rated health, poor mental health (K6 > = 5) at follow-up (cycle 8 in 2008/09) among respondents aged 15–17 years old (n = 250) from the Statistics Canada’s National Population Health Survey (NPHS)

	**Model 1**		**Model 2**		**Model 3**	
Controlling for:	Sex, age, and frequency of fruit and vegetable intake at baseline		Covariates controlled at Model 1+ highest level of education in the household		Covariates controlled at Model 2 + the other covariates† at baseline	
	OR	95% CI	OR	95% CI	OR	95% CI
Self-rated health						
Fruit and vegetable consumption = Low (<5)‡	1.00		1.00		1.00	
Fruit and vegetable consumption = High (> = 5)‡	0.36	(0.16-0.81)	0.32	(0.15-0.71)	0.29	(0.13-0.69)
p for difference (df = 1)	0.014		0.005		0.005	
Mental health (K6 scores)						
Fruit and vegetable consumption = Low (<5)‡	1.00		1.00		1.00	
Fruit and vegetable consumption = High (> = 5)‡	1.11	(0.51-2.44)	1.06	(0.48-2.35)	1.11	(0.48-2.54)
p for group difference (df = 1)	0.791		0.888		0.813	

OR, odds ratio; CI, confidential interval.

†:The other covariates included alcohol consumption, smoking status, BMI, physical activity, and household size.

‡:Frequency per day. The numbers of respondents were 160 and 90 for the Low and High groups, respectively.

## Discussion

The findings of this study indicated that high consumption of fruits and vegetables in adolescence had a positive impact on self-rated health, but not on mental health, in early adulthood among participants of the Statistics Canada’s National Population Health Survey. The present study demonstrated a longitudinal association between high fruit and vegetable consumption in adolescence and excellent self-rated health in early adulthood. High fruit and vegetable consumption in adolescence was also marginally significantly associated with better self-rated health when a different categorization of self-rated health was used. With a smaller sample that provided information on household income, high fruit and vegetable consumption in adolescence was not significant, but was slightly associated with better self-rated health, which is possibly attributable to a smaller statistical power. The findings were consistent with previous cross-sectional findings that observed the positive relationships between fruit and vegetable consumption and self-rated health among adolescents [26-28] and early-adults [10]. This study added new longitudinal findings on this association to expand the already accumulated evidence. The findings were also in agreement with previous findings from observational studies on adults and elderly [11-13] and with an intervention study that reported improvement in health-rated quality of life, which was associated with increased fruit and vegetable consumption among adults [8]. Thus, these findings suggest that fruit and vegetable consumption in adolescence affects self-rated health in the early adulthood.

Self-rated health may have physiological and cognitive components. One’s perception of his or her health is affected by having overt diseases, risk factors, lowered functioning, and bodily sensation [9]. Fruits and vegetables supply dietary fiber, and fiber intake is linked to lower incidence of cardiovascular disease [42], diabetes [43], cancer [44,45], and obesity [46]. Fruits and vegetables also supply vitamins and minerals to the diet and are sources of phytochemicals that function as antioxidants, phytoestrogens, anti-inflammatory agents, and other protective mechanisms [47-50]. Such biological benefits of fruit and vegetable consumption could prevent chronic diseases such as cardiovascular diseases or their risk factors [3-7], thus, may enhance one’s perception of his or her health. Self-rated health also includes a cognitive judgment of one’s health status compared to reference groups, past experiences, and cultural norms [9]. High consumption of fruits and vegetables may serve to develop greater self-efficacy in controlling health, which may lead to the perception of better health. Possible mechanisms that underlie the association between fruit and vegetable consumption and better self-rated health should be investigated in more detail with attention to the multidimensional aspects of self-rated health. It might be interesting to note that in this study, high fruit and vegetable consumption was associated with an excellent or non-excellent distinction of self-rated health compared to a better or poor distinction. In other words, fruit and vegetable consumption was more closely linked with the top 25% of self-rated health than it was with the bottom 25%. Therefore, fruit and vegetable consumption may be associated with promoting a higher end of physical functioning rather than preventing poor health status.

The association of fruit and vegetable consumption and self-rated health became clearer when the latter classification (secondary one) was used. This classification was based on the recommended minimum consumption of fruit and vegetables. Thus, meeting this may have a valuable impact on promoting self-rated health.

The present study failed to show a longitudinal association between fruit and vegetable consumption and mental health when mental health was measured via psychological distress during adolescence and early adulthood. Previous studies with adults have shown that vitamins are associated with better mental health [14-18]. However, a certain number of studies have reported no associations between fruit and vegetable consumption and mental health [8,19,20]. As such, fruit and vegetable consumption may not be associated with better mental health during adolescence and early adulthood. A possible reason of the lack of association between fruit and vegetable consumption and mental health is that fruit and vegetable consumption is too rough an indicator of vitamin intake. It is also possible that other factors such as meal preparation, dining environment, social interaction, and cultural aspects of fruit and vegetable consumption may mask this association [19]. It is unclear from this study why participants who consumed fruits and vegetables frequently had poorer mental health. A possible reason is that consuming fruits and vegetables frequently may be associated with less consumption of other nutritional elements that are relevant to incidences of depression and anxiety, such as fish oil or omega 3 fatty acid [51]. It is also possible that the lack of significant association might be due to the limited age composition of the sample, because most previous studies which reported positive association between vitamins and mental health were conducted in samples with a broader age range [14-18].

Several limitations of this study should be noted. First, the results of the current study may have been influenced by those who did not participate in the study. Those lost to follow-up were more likely to be male, less educated, and having less healthy lifestyle including lower consumption of fruits and vegetables, compared to those who completed the follow-up. The association may have been underestimated if respondents with low consumption of fruits and vegetables and poor health were more likely to be lost to follow-up. In addition, the generalization of the results may be limited because the sample was more represented by females, higher socioeconomic position, and healthier lifestyles. To avoid such bias or limited generalization, it might have a merit to perform a large-scale cross-sectional analysis on this type of population-based health behavior survey, in addition to a prospective analysis like the present study. Second, the classification of health outcome variables in this study was arbitrary. Other ways of categorization may result in different findings. Third, while it was well validated as a proxy measure of fruit and vegetable consumption, the questionnaire used to measure fruit and vegetable consumption in this study did not quantitatively estimate the consumption. However, the use of a validated, but long food frequency questionnaire is not always practical in population surveys. Fourth, although we conducted analyses that adjusted for several covariates, other potential factors, such as dietary culture and family norms on diet, might have confounded the results. Finally, the sample size may have been too small to detect potential associations between fruit and vegetable consumption and health outcomes. A larger sample size might be needed.

## Conclusions

In conclusion, this longitudinal study indicated that fruit and vegetable consumption in adolescence had a beneficial influence on self-rated health in early adulthood. This finding expands already accumulated evidence in adults by adding longitudinal evidence from adolescents. A future investigation may be promising if it is designed as a larger, longer longitudinal study that adjusts for a wide range of possible confounding factors such as genetic and cultural factors. As more evidence is accumulated, it is recommended that stakeholders develop a policy to encourage adolescents’ consumption of fruits and vegetables to ensure better health in the future.

## Competing interests

The authors declare that they have no competing interests.

## Authors’ contributions

YT conceived the study, did literature search, designed the study, accessed to data, analyzed and interpreted data, and drafted the manuscript. NK did literature search, designed the study, analyzed and interpreted data, and provided critical advices to the manuscript. Both authors read and approved the final manuscript.

## Pre-publication history

The pre-publication history for this paper can be accessed here:

http://www.biomedcentral.com/1471-2458/13/1206/prepub
